# Floral resource partitioning by individuals within generalised hoverfly pollination networks revealed by DNA metabarcoding

**DOI:** 10.1038/s41598-018-23103-0

**Published:** 2018-03-23

**Authors:** Andrew Lucas, Owen Bodger, Berry J. Brosi, Col R. Ford, Dan W. Forman, Carolyn Greig, Matthew Hegarty, Laura Jones, Penelope J. Neyland, Natasha de Vere

**Affiliations:** 1 Department of Biosciences, College of Science, Swansea University, Singleton Park, Swansea, SA2 8PP Wales UK; 2 Institute of Life Science, School of Medicine, Swansea University, Singleton Park, Swansea, SA2 8PP Wales UK; 3 Department of Environmental Sciences, Emory University, 400 Dowman Drive NE, Atlanta, Georgia 30322 USA; 4 National Botanic Garden of Wales, Llanarthne, Carmarthenshire, Wales SA32 8HG UK; 5 Institute of Biological, Environmental and Rural Sciences, Aberystwyth University, Aberystwyth, SY23 3EE Wales UK

## Abstract

Pollination is a key ecosystem service for agriculture and wider ecosystem function. However, most pollination studies focus on Hymenoptera, with hoverflies (Syrphidae) frequently treated as a single functional group. We tested this assumption by investigating pollen carried by eleven species of hoverfly in five genera, *Cheilosia*, *Eristalis*, *Rhingia*, *Sericomyia* and *Volucella*, using DNA metabarcoding. Hoverflies carried pollen from 59 plant taxa, suggesting they visit a wider number of plant species than previously appreciated. Most pollen recorded came from plant taxa frequently found at our study sites, predominantly Apiaceae, Cardueae, *Calluna vulgaris*, *Rubus fruticosus* agg., and *Succisa pratensis*, with hoverflies transporting pollen from 40% of entomophilous plant species present. Overall pollen transport network structures were generalised, similar to other pollination networks elsewhere. All hoverfly species were also generalised with few exclusive plant/hoverfly interactions. However, using the Jaccard Index, we found significant differences in the relative composition of pollen loads between hoverfly genera, except for *Volucella*, demonstrating some degree of functional complementarity. *Eristalis* and *Sericomyia* species had significant differences in relative pollen load composition compared to congeners. Our results demonstrate the range of pollens transported by hoverflies and the potential pollination function undertaken within this ecologically and morphologically diverse guild.

## Introduction

Pollination is a key ecosystem service which sustains significant food production^1,2^. In addition, by enabling wild plant reproduction^3^, pollination maintains plant diversity and therefore promotes wider ecosystem functioning on which other services, such as production of biomass and the cycling of nutrients, depend^4^.

Understanding interactions between wild pollinators and plants is critical, because pollination network structure has implications for the stability of pollination as an ecosystem service in the face of environmental change^5–7^. Pollination networks previously studied have had a generalised structure, with plants having numerous potential pollinators, and pollinators in turn visiting many plant species^8,9^. The plant species in these networks are predominantly ecological and functional generalists, with flowers that are accessible to a range of potential pollinators^10,11^. Such generalised networks can be more robust to species extinctions, because plants are able to exchange pollinator species if pollinator populations fluctuate^12^.

There is an increasing appreciation that flies (Diptera) have an important role in pollination, particularly at higher latitudes^13,14^. Amongst the Diptera, hoverflies (Syrphidae) are key pollinators of both crops^15^ and wild plant species^16^. Most studies of hoverflies have focussed on a small number of well-known species, particularly *Eristalis tenax* and *Episyrphus balteatus*^17,18^. However, hoverflies are a diverse family containing approximately 6000 species worldwide^19^, with 282 species recorded from Britain^20^. The mouthparts of arthropod species that feed on pollen show a range of morphological specialisation^21^. Mouthpart length in hoverflies has been shown to have some influence on flower selection^22,23^, which could in turn affect pollination network structure^24^. The flower preferences of adult hoverflies, and their role in pollination, are little known for most species^25^. With the observed decline in many hymenopteran pollinators, there is a need for a greater understanding of the role of flies in plant – pollinator interactions^26^.

A range of techniques have been used to study plant – pollinator interactions. There is a long history of counting plant visitors in natural communities^27,28^, whilst other methods include devising experimental situations with a limited choice of foraging options^29^, or retrieving and identifying pollen from insect guts using traditional palynological methods^23^. However, the existing techniques have limitations. Hoverflies can be difficult to follow in the wild, with some species known to forage in woodland canopies^30^. The visual identification of pollen can also be challenging, even for experienced observers, given the similarity in pollen morphology within some plant families^31^.

DNA metabarcoding – the sequencing and identification of mixed DNA samples using next-generation sequencing – has opened new opportunities for study in a range of ecological contexts^32^. This technique has been used to investigate the composition of invertebrate communities^33^ and to examine the structure of food webs^34,35^, and also has shown considerable potential in the study of pollen transport^36–38^. DNA metabarcoding has been shown to be a reliable method of identifying pollen, either carried as loads by insects^39^ or derived from honey^31^. Molecular techniques to identify pollen have been used to investigate wild bee pollination of native and non-native plant species^40^, and pollen collection by domestic honey bees^41^.

Here, we use DNA metabarcoding to investigate pollen transport in hoverfly communities in fen-meadows, a species-rich grassland community found on peaty mineral soils in grasslands of conservation importance in lowland Wales, United Kingdom^42^, and which are an endangered habitat of European importance^43^. Such agriculturally unimproved grasslands remain a significant part of the biodiversity in south-west Wales^44^, and have the potential to provide ecosystem services, such as pollination by hoverflies, to the wider countryside^45^.

We retrieved pollen carried by eleven hoverfly species in five genera – *Cheilosia*, *Eristalis*, *Rhingia*, *Sericomyia* and *Volucella* – and sequenced the standard plant DNA barcode region *rbcL*. We then matched and identified the sequences using a standard pre-existing library of plant barcode sequences^46^, allowing us to characterise the overall composition of pollen loads for each hoverfly species. We used this information to construct pollen transport networks for the three grasslands in our study, and calculated a series of established network metrics to describe structure at the level of the overall network (*H*_2_’) and species (*d’*)^47^.

We predicted that the networks would have a generalised structure (i.e. low values of *H*_2_’ and *d’*) consistent with other networks studied elsewhere^48,49^. Using the Jaccard Index, we investigated the similarity in pollen load composition between the five genera, and between species in two genera, *Eristalis* and *Sericomyia*, where more than one species was available. Given the notable morphological and behavioural differences, we predicted significant differences in the pollen loads between these distinctive hoverfly genera. For the six *Eristalis* species we predicted that, given that all species are common in the study area and are relatively morphologically uniform, there would be no differences in the composition of pollen loads between species. However, the two *Sericomyia* species are quite distinctive in their morphology and ecology. *Sericomyia silentis* is a relatively common, wasp-mimic species, whilst *S*. *superbiens* is a bumble bee mimic that, in Britain, is mainly restricted to wet pastures in the west and north. We therefore predicted that, in contrast to *Eristalis* species, there would be significant differences in the composition of pollen loads between the two species of *Sericomyia*.

## Results

### Overview

**Figure 1:**
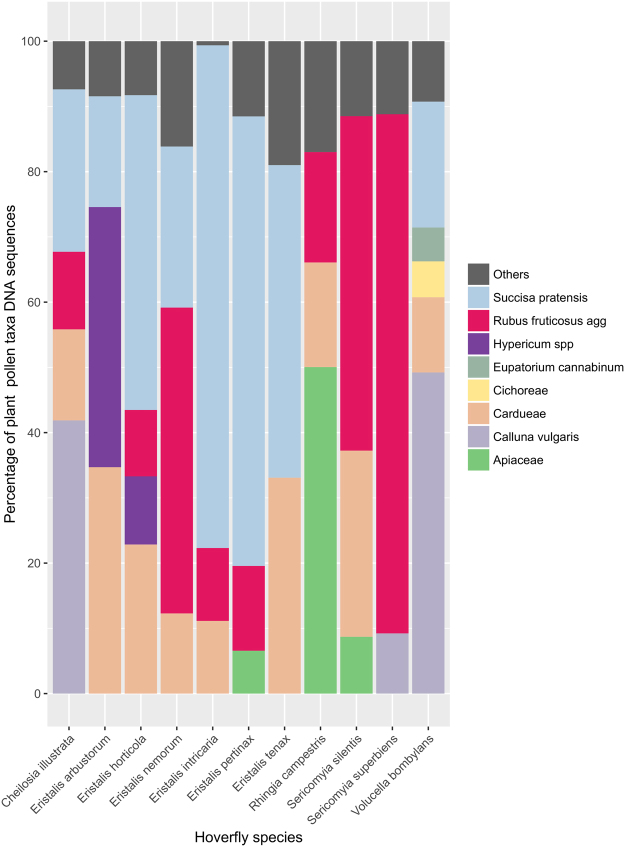
Mean % of plant taxa pollen DNA sequences retrieved from pollen loads carried by 11 hoverfly species at three grasslands in west Wales, July–August 2014. For clarity, all plant taxa contributing less than 5% of sequences for a hoverfly species have been combined as ‘Others’.

**Figure 2:**
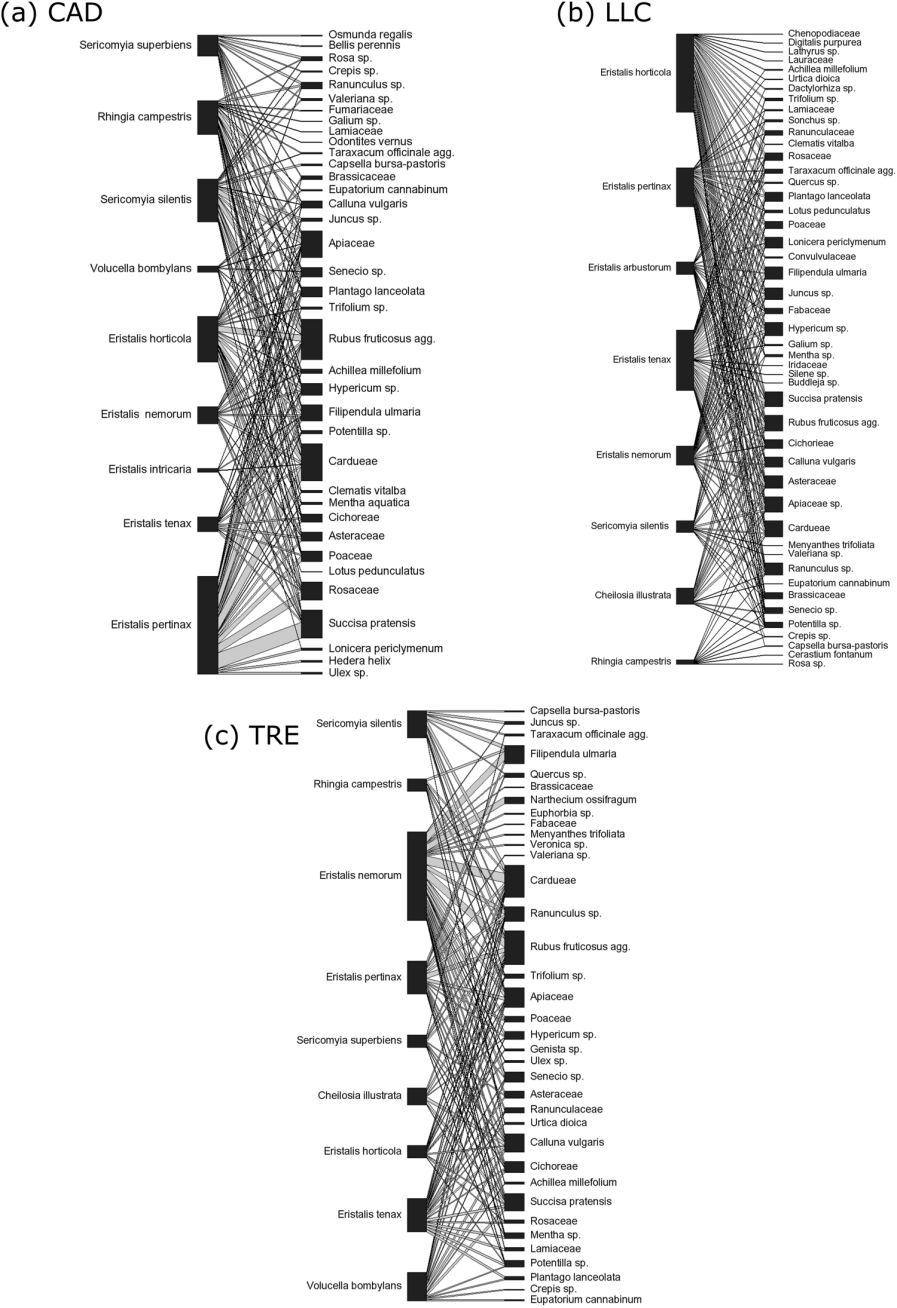
Pollen transport network derived from analysis of pollen carried by hoverflies at site CAD (top left) LLC (top right) and TRE (bottom centre), July–August 2014. The length of the hoverfly and plant taxon bars indicates the proportion of the respective taxa in the study, and the width of the connecting ribbon represents the strength of the interaction.

We sequenced pollen loads from 143 hoverflies of 11 species (*Cheilosia illustrata, Eristalis arbustorum, E. horticola, E. intricaria, E. nemorum, E. pertinax, E. tenax, Rhingia campestris, Sericomyia silentis, S. superbiens*, and *Volucella bombylans*) (Table 1 and Fig. 1). A total of 1,810,674 sequences over 450 bp in length could be attributed to tagged sequences of *rbcL*. Of these, 1,791,574 (98.9%) could be identified to plants at species, genus or family level. We identified 58 plant taxa from pollen retrieved from all 11 hoverfly species, consisting of 21 species, 22 genera and 15 families (Figs 1 and 2) (Supplementary information ST3). Plant species richness (Table 1) was comparable at each site, ranging from 64 to 75. A list of the plant species recorded at each site is given in Supplementary Table ST2.

**Table 1:** Values of H_2_’, plant species richness, entomophilous plant species richness, number of entomophilous plants in hoverfly pollen, number of pollen taxa, and values of d’ for pollen loads carried by 11 species of hoverfly at three grassland sites in west Wales, July–August 2014.

Species	CAD			LLC			TRE			Mean d'	Stand. Dev.	Total n Hoverflies
H_2_’	0.19			0.12			0.24					
Site plant species richness	64			75			67					
Entomophilous plant species richness	33			39			31					
Entomophilous plant species also present in hoverfly pollen	13			17			13					
	**No**. **Pollen Taxa**	**d'**	**n**	**No**. **Pollen Taxa**	**d'**	**n**	**No**. **Pollen Taxa**	**d'**	**n**			
*Cheilosia illustrata*			0	16	0.23	6	8	0.17	3	0.20	0.04	**9**
*Eristalis arbustorum*			0	22	0.09	2			0			**2**
*Eristalis horticola*	24	0.08	9	38	0.04	11	13	0.19	1	0.10	0.08	**21**
*Eristalis intricaria*	7	0.00	1			0			0			**1**
*Eristalis nemorum*	12	0.09	3	28	0.03	3	31	0.14	8	0.09	0.03	**14**
*Eristalis pertinax*	26	0.12	25	31	0.04	6	17	0.09	4	0.08	0.04	**35**
*Eristalis tenax*	12	0.10	3	33	0.06	10	21	0.17	2	0.11	0.03	**15**
*Rhingia campestris*	24	0.19	8	12	0.37	1	8	0.15	2	0.26	0.12	**11**
*Sericomyia silentis*	23	0.21	14	17	0.19	5	12	0.24	5	0.21	0.03	**24**
*Sericomyia superbiens*	16	0.32	5			0	11	0.12	2	0.22	0.14	**7**
*Volucella bombylans*	10	0.15	1			0	15	0.20	3	0.18	0.04	**4**
Total n			69			44			30			**143**

The proportions of sequences (percentages) for each plant taxa recovered varied between hoverfly species. The sequences contributing 5% or more for a species are shown in Fig. 1, whilst network Fig. 2 shows all plant pollen taxa recovered from hoverflies at each site. Pollen sequences from *Cheilosia illustrata* were predominantly from *Calluna vulgaris* and *Succisa pratensis*, with lesser amounts of *Rubus fruticosus* agg. and Cardueae. *Eristalis* species carried a number of pollen taxa, with sequences from the Cardueae, *Rubus fruticosus* agg., and *S*. *pratensis* predominating, although *Hypericum* species were also a significant proportion of the pollen load on *E*. *arbustorum* and, to a lesser extent, *E*. *horticola*. Apiaceae were the most frequently recorded sequences carried by *Rhingia* *campestris* with smaller amounts of both Cardueae, *Rubus fruticosus* agg. and Brassicaeae. Both *Sericomyia*. *silentis* and *S*. *superbiens* sequences came predominantly from *Rubus fruticosus* agg. pollen. However, *S*. *silentis* also favoured Cardueae and, to a lesser extent, Apiaceae, whilst *S*. *superbiens* had smaller amounts of both *Calluna vulgaris* and *Succisa pratensis*. *Volucella bombylans* pollen sequences consisted mainly of *Calluna vulgaris*, *Succisa pratensis* and Cardueae, with smaller amounts of Cichoreae and *Eupatorium cannabinum*.

The number of entomophilous plant species at each site, and the number of those species that were also recorded in pollen samples from all hoverflies is also shown in Table 1. The proportion of entomophilous species at each site also recorded as present in pollen were 13/33 (39%) at site CAD, 17/39 (44%) at site LLC, and 13/31 (42%) at site TRE.

**Table 2:** Comparison of the pollen loads of five hoverfly genera using the Jaccard Similarity Index. One species was tested for *Cheilosia*, *Rhingia* and *Volucella*, and therefore the species name is given. The analysis used the Dunn–Šidák correction for multiple comparisons. The *p*-value significance cut-off is 0.0073008.

Species/Genus	F	R2	Unadjusted *p* value
*Cheilosia illustrata*	5.895	0.04	0.0001*
*Eristalis* species	15.568	0.099	0.0001*
*Rhingia campestris*	7.147	0.048	0.0001*
*Sericomyia* species	16.24	0.103	0.0001*
*Volucella bombylans*	3.115	0.022	0.013

### Pollination Network Structures

The network metrics are given in Table 1, with the networks themselves illustrated in Fig. 2. The network specialisation metric *H*_2_’ (Table 1) is a measure of overall generalisation or specialisation of a network, and ranges from 0 (perfect generalisation) to 1 (perfect specialisation). The values of *H*_2_’ for the networks at each site in our study had values below 0.5, ranging from 0.12 (LLC) to 0.24 (TRE). This indicates that the pollen transport networks at the site level were more generalised in their structure than specialised^47^, with plants having multiple hoverfly species transporting their pollen, and hoverfly species in turn visiting multiple plant species (see also Fig. 2).

The network metric *d’* (Table 1) measures the degree of exclusivity in a species’ interactions in a network, and ranges from 0 (no exclusivity) to 1 (complete exclusivity). The range of values of *d’* for all *Eristalis* species combined were relatively low (mean = 0.08, range 0–0.19) indicating that few interactions were unique to these species. However, results amongst other species had some higher values of *d’*, notably for *Rhingia campestris* (median = 0.19, range 0.15–0.37). However, a Kruskall – Wallis test of all values of d’ showed no significant difference between species (X^2^ = 16.23, *p* = 0.09). The number of exclusive interactions by species was therefore low, with no difference in exclusivity between species. The generalisation of whole networks and the lack of exclusive interactions at the species level are further illustrated by the network diagrams for each site shown in Fig. 2. These show all plant pollen taxon groups that were identified as part of this study at each site, and the presence or absence of pollen taxa carried by each hoverfly species.

### Pollen Load Differences – Genera and Species

Having found very few exclusive interactions, we then tested for differences among taxa in pollen load composition, as measured by mean pairwise comparisons between individuals using the Jaccard Similarity Index. We initially investigated whether a significant difference existed between pollen loads of the five hoverfly genera in this study, using an *adonis* (permutational MANOVA) analysis. We found that there is a significant difference between hoverfly genera overall (F_(4,142)_ = 9.860, R^2^ = 0.222, *p* < 0.001).

To explore specifically where these differences were, we then ran five separate analyses comparing each genus with the remaining four genera. We used the Dunn–Šidák correction to correct for multiple comparisons, yielding a *p*-value significance cut-off of 0.0073008. We found that each genus carried pollen loads with significantly distinct species composition, compared to all other hoverfly genera, with the exception of *Volucella* which was not significant when correcting for multiple comparisons (Table 2).

At the species level, in an initial analysis comparing all eleven species, there was a significant difference in pollen load composition (F_(10,142)_ = 6.335, R^2^ = 0.324, *p* = 0.0001). We then investigated whether there were significant differences in pollen load composition between the species in the two genera for which there were multiple species. For the six *Eristalis* species, there was a significant difference in pollen load composition (F_(5,87)_ = 2.972, R^2^ = 0.153, *p* = 0.033). This was contrary to our prediction of no significant difference. For the two *Sericomyia* species there was also a significant difference in pollen loads (F_(1,30)_ = 3.695, R^2^ = 0.113, *p* = 0.016). This confirmed our prediction of a significant difference in pollen loads between species.

## Discussion

The eleven hoverfly species in this study form a pollinator community that is relatively generalised, both in terms of overall network structure and individual species. Although a large number of taxa were recorded from pollen removed from hoverflies, there were few exclusive hoverfly/pollen taxa interactions, with the main constituents of the pollen loads of all species being relatively similar. Nonetheless, there were significant differences in the composition of pollen loads among hoverfly species, which suggests they may fulfil complementary roles in pollen transport in the grassland habitats we studied.

A relatively generalised structure is a consistent feature of pollinator visitation networks found in other ecosystems, such as arable habitats^50^, heathlands^48^, urban areas^49^, and Mediterranean grasslands^51^. In these systems, plants have a number of pollinators, and pollinators, in turn, visit a number of different plant species. The pollen transport networks of hoverfly communities at our sites showed a similar pattern, with *H*_2_’ values lower than 0.5, indicating a structure closer to generalisation than specialisation.

The network metric *d’* measures the degree of specialisation, in terms of exclusive interactions, at the species level. Values of *d’* were especially low for the *Eristalis* species, indicating low levels of specialisation. This was unsurprising given the morphological similarity of the species in this genus, which have been noted visiting a comparable suit of plant species^52^. The slightly higher values of *d’* amongst non-*Eristalis* hoverfly species is of note, particularly that of *Rhingia campestris* which may indicate a small degree of exclusivity in their pollen loads^47^. However, values of *d’* are still less than 0.5, and there was no significant difference in the values of *d’* between species. Overall, pollen transport networks amongst hoverflies at our sites were characterised by generalisation at the species level. Values of *d’* for hoverflies in grassland habitats based on observational studies range from 0.2 to 0.33^53,54^. The values of *d’* in this study were lower (range of mean *d’* values 0.08–0.26), which may reflect the ability of DNA metabarcoding to detect a greater range of plant – insect interactions.

Differences in the value of *d’* reflect the degree of exclusive interactions of a particular species. Hoverflies at our sites carried pollen predominantly drawn from a suite of 8 plant taxonomic groups (Apiaceae, *Calluna vulgaris*, Cardueae, Cichoreae, *Eupatorium cannabinum*, *Hypericum* species, *Rubus fruticosus* agg. and *Succisa pratensis* (Fig. 1), many of which were visited by multiple hoverfly species. However, there were significant differences in interspecific pollen loads between all genera in our study, with the exception of *V*. *bombylans*, and between species in the genera *Eristalis* and *Sericomyia*. This may reflect some degree of floral resource partitioning by adult hoverflies at our sites.

Previous work gives some limited evidence of niche partitioning in hoverflies^16^. Temporal niche segregation has been observed in adult *Copestylum* hoverflies in desert environments^55^, and Fründ *et al*.^53^ found evidence of differences in flower preference in four species of adult hoverflies: *Episyrphus balteatus,* *Eristalis tenax*, Sy*ritta pipiens* and *Sphaerophoria scripta*. Resource partitioning has also been previously noted in a range of pollinators, including bumblebees^56^, mixed bee communities^57^, birds^58^ and bats^59^. Our results demonstrate significant differences in the proportions of pollen taxa in the pollen loads of hoverfly genera and species, suggesting that hoverfly species may be fulfilling a complementary role to each other in pollen transport in the grasslands in our study.

A number of processes may be leading to differences in pollen loads between species. These include innate differences in flower choice^60^, agonistic interactions between hoverfly species, or interactions between hoverflies and other pollinator guilds^61,62^. Plant species can vary in the energy content of their nectar^63^, and there is some limited evidence of differences in hoverfly metabolism between species^64^. There is also a wide variation in the proboscis length in the species in this study^65^, ranging from 3.3 mm (*Cheilosia illustrata*), to 10.6 mm (*Rhingia campestris*) which could result in differences in plant visitation. Differences in the composition of pollen loads may therefore be arising from a combination of morphology, differing physiological requirements between species, behaviour, and interaction with other pollinator species. Understanding the mechanisms behind resource partitioning in hoverfly pollen loads is critical, because pollinator species diversity is a key factor in the functioning of pollination as an ecosystem service^66,67^.

Overall, 59 plant pollen taxa were recorded on hoverflies at our field sites, some of which will include several species (e.g. ‘Apiaceae’). Morris^68^, lists 188 plants visited by all hoverfly species in Surrey in southern England. Our study, based on eleven species in three fields in west Wales, suggests that the range of plants visited by hoverflies is far wider than previously appreciated. Hoverflies were transporting pollen from between 39% and 44% of entomophilous plant species recorded at our sites. This result should be interpreted with care, because some entomophilous plant species (e.g. *Prunus spinosa* and *Crategus monogyna*) would not have been flowering at the time of the study. Hoverflies also carried pollen from anemophilous species, such as grasses. Pollen from such species has been found in the diet of some hoverflies^69^ and bee pollen loads^41^, although whether this constitutes a significant role pollination function is unclear^60^. In addition, the data used to compile the plant species list was originally collected to characterise the grassland communities, and not to provide an exhaustive site species list. However, hoverflies are potentially transporting the pollen from a significant proportion of the entomophilous plant community on our sites. Further work is needed, combining pollen load information with contemporaneous data on plant flowering, to fully assess the potential contribution of hoverflies to pollination.

The pollen loads for *Cheilosia illustrata* in this study are notable for the scarcity of *Heracleum sphondylium*, as the larvae feed exclusively on this species^70^. Observations of other *Cheilosia* species have shown this genus visits a variety of species^71^. Our results for *C*. *illustrata* recorded a range of plant species, including a small amount of Apiaceae pollen, which may represent *H*. *sphondylium*. The genus *Eristalis* (principally *E*. *tenax*) is one of the most well-studied hoverfly genera, recorded visiting a range of plant species^72^. In this study, the pollens most frequently recovered from *Eristalis* species were *S*. *pratensis*, *R*. *fruticosus agg*., and Cardueae species. The pollen loads of *R*. *campestris* were of particular interest, as the genus has long mouth parts, making many tubular flowers accessible to this species^73^. However, loads on this species were distinctive for their high proportion of Apiaceae pollen, a plant family with open, readily accessible inflorescences utilised by a number of hoverfly species^16^. The species *S*. *silentis* and *S*. *superbiens* have differences in status in Britain; *S*. *silentis* is relatively common, whilst *S*. *superbiens* is a localised species primarily found in the west and north^74^. *Sericomyia silentis* has been noted as a visitor to a number of plant species, including Ericaceae, Asteraceae and Apiaceae^68^. In Estonia, *S*. *superbiens* fed on *Centaurea* and *Sonchus* species^75^. In this study, the pollen loads of both species consisted mainly of *R*. *fruticosus*. The final species, *V*. *bombylans*, has been observed visiting domestic *Rubus* species^76^, whilst other *Volucella* species have been recorded visiting a range of flowers, including *Calluna vulgaris*^77^. In our study, it was the only species where the highest percentage of pollen carried came from *Calluna vulgaris* together with *S*. *pratensis*.

Numerous authors have noted that pollen transport by an insect does not imply that it is an effective pollinator^10,78,79^. Networks may appear to be generalised due to a large number of potential pollinators visiting plants, but this can resolve into a much more specialised network when pollination effectiveness, as opposed to pollen transport, is taken into account^48^. Our results demonstrate the role of hoverflies in pollen transport in grassland ecosystems. However, further study is required to evaluate whether the pollen transport we have observed in this study is translating into successful pollination, and to more fully describe hoverfly foraging ecology.

This study was concerned with eleven hoverfly species that are, with the exception of *R*. *campestris*, relatively large wasp or bee mimics^70^. However, hoverflies are a diverse family, which in Britain includes small species, such as the genus *Neoascia*^80^ or species that are morphologically uniform with little or no mimicry of hymenoptera, such as many *Cheilosia* species^71^. Moreover, this study was limited to a short period in late summer at three sites. The evidence of resource partitioning found amongst the hoverfly species in our study, and of involvement with a high proportion of plant taxa invites further investigation to determine how the full diversity of the hoverfly fauna contributes to pollination. Understanding the full role of hoverflies in plant pollinator interactions is important because flower visitation by hoverflies may be complementary to bees, taking place when the resource is unavailable to other pollinators, not least because air temperature can influence both insect activity and nectar concentration^60^. These seasonal and diel effects may have a key influence on flower visitation and potential pollination by hoverflies^81^. Pollination networks can also vary in their structure between years, so that observation over a number of field seasons is required to fully describe how insect communities deliver a pollination ecosystem service^82^.

Similarly, further examination is required of the role of hoverflies in the function of grassland systems. Whilst most pollen carried by hoverflies was from a small number of common plant taxa, these plants may be supporting diverse pollinator communities that, in turn, can occasionally pollinate scarce plant species^83^. The many pollen taxa carried by hoverflies at low levels may represent a significant role in the pollination of scarcer plant species, and thus in wider ecosystem function.

This study demonstrates that, whilst there is some overlap in the pollen taxa transported by hoverfly species, there are differences in the composition of pollen loads between species, which may imply that that hoverfly species are performing subtly different pollination functions. Although it may not always be practical to identify hoverflies to species in observational field studies, this specialisation should be considered when assessing the value of management interventions for pollinators. This study is one of the first to use DNA metabarcoding to investigate a Dipteran pollinator community, and adds to our understanding of the role of hoverflies in pollen transport and the functioning of conservation grassland habitats.

## Methods

### Site Descriptions

The study took place during 2014 at three grassland sites of high conservation importance in west Wales, United Kingdom. We collected hoverflies at these locations (referred to here as ‘CAD’, ‘LLC’, and ‘TRE’), where the National Vegetation Classification community M24 *Molinia caerulea – Cirsium dissectum* fen-meadow (*Cirsio – Molinietum caerulae*)^42^ was present (for full site information, see Supplementary Information Table ST1).

All sites were typical of this community. *Molinia caerulea*, *Potentilla erecta*, *Succisa pratensis*, and *Lotus pedunculatus* were all common in the sward, with *Cirsium dissectum* occurring more locally. Other frequent forb species included *Calluna vulgaris*, *Ranunculus* species, other *Cirsium* species, *Serratula tinctoria* and *Carum verticillatum*. Sward height was between approximately 20 cm and 60 cm. Each site consisted of a single field, surrounded by hedgerows consisting mainly of *Crategus monogyna*, *Prunus spinosa*, and *Corylus avellana*, with frequent *Rubus fruticosus* agg. also present. We complied a plant species list from existing grassland quadrat data for each site^84^, together with records of hedgerow species for each site collected in October 2015. From this species list, a sub-list of entomophilous plants was created by excluding grass (Poaceae), sedges (Cyperaceae), and rushes (Juncaceae). Subsequently, a list of all plant pollen taxa recorded from pollen loads of all hoverfly species was compared to the entomophilous plant sub-list. For this purpose, all plant taxa recorded at a higher level than species were assumed to have come from a single plant species, irrespective of how many plant species were present. A species list for each site is included as Supplementary Table ST2.

### Field Collection of Hoverflies

We collected insects between July 10 and August 27 2014. To ensure the insects captured were representative of the site as a whole, a series of transects 20 m apart were walked across each site, during which hoverflies were collected, ensuring that the entire site was searched. Each site was visited twice during the study period, between 11:00 and 15:30, and searched for three hours in total, with transects repeated as necessary. We placed the insects individually in 1.5 ml tubes immediately after capture, which were subsequently stored at −20 °C prior to pollen removal. Following pollen removal, we identified the hoverflies morphologically to species^70^.

### Pollen removal

We initially washed the insects in the tube in which the insect had been placed in the field. The fly was immersed in 1 ml of a 1% sodium dodecyl sulphate (SDS) and 2% poly-vinyl pyrrolidinone (PVP) solution in water. The tube was shaken vigorously by hand for 1 minute, and then centrifuged briefly to ensure that the insect was fully immersed in the liquid. It was then allowed to stand at room temperature for 5 minutes. The tube was then shaken vigorously by hand for 20 seconds. The fly was removed using forceps to a clean 1.5 ml microfuge tube and frozen at −20 °C for subsequent species identification. The tube containing the detergent and pollen was centrifuged at 13 000 rpm for 5 minutes.

### DNA extraction

We used the DNeasy plant mini kit (Qiagen) for DNA extraction. The supernatant was discarded and the pellet suspended in 400 μL AP1 and 80 μL proteinase K (1 mg/ml). This was incubated for 60 minutes at 65 °C in a water bath and then disrupted using a TissueLyser II (Qiagen) for 4 minutes at 30 Hz with 3 mm tungsten carbide beads. The subsequent steps were followed according to the manufacturer’s instructions, with the exception that QIAshredder column and second wash stage were omitted.

### Amplification and Sequencing: Illumina Miseq

We amplified the DNA using the *rbcL* DNA barcode marker region^85^. Two rounds of PCR were carried out: a primary tailed amplification of the *rbcL* region, followed by a second round of amplification that added the Illumina Nextera index adaptor sequences so that samples could be processed on Illumina platforms and be subsequently separated via bioinformatic processing. Samples were initially amplified using the universal primers *rbcLaf* and *rbcLr506*^46^, to which adaptor 5′ overhangs had been added:

(*rbcL*af + adaptor: TCGTCGGCAGCGTCAGATGTGTATAAGAGACAGATGTCACCACAAACAGAGACTAAAGC

*rbcL*r506 + adaptor: GTCTCGTGGGCTCGGAGATGTGTATAAGAGACAGAGGGGACGACCATACTTGTTCA).

PCR was performed using a final volume of 20 μl. A mix of 10 μl of 2× Phusion Mastermix (New England Biolabs), 0.4 μl of 5 µM forward primer (*rbcL*af+adaptor), 0.4 μl of 5 µM reverse primer (*rbcL*506+adaptor), and 7.2 μl of molecular biology grade water was made, to which 2.0 μl of template DNA was added. The PCR conditions were: 95 °C for 2 minutes; 95 °C for 30 seconds, 50 °C for 1 minute 30 seconds, 72 °C for 40 seconds (35 cycles); 72 °C for 5 minutes, 30 °C for 10 seconds. We visualised the PCR products using agarose gel electrophoresis to confirm successful amplification. This process was repeated three times, and the PCR products combined, to account for possible bias in any one PCR.

Products from the first PCR were purified following IIlumina’s 16S Metagenomic Sequencing Library Preparation protocol^86^ using Agencourt AMPure XP beads (Beckman Coulter). The Index PCR stage (following the Illumina protocol) used a 25 µl reaction (12.5 μl of 2× Phusion Mastermix, 2.5 μl of Nextera XT i7 Index Primer, 2.5 μl of Nextera XT i5 Index Primer, 5 μl of PCR grade water, and 2.5 μl of purified first-round PCR product). PCR clean-up 2 of the Illumina protocol was then followed, cleaning 20 µl of Indexed PCR product, with a 1:0.8 ratio of product to AMPure XP beads.

Amplified products were quantified using a Qubit fluorescence spectrophotometer (Life Technologies) and pooled at equal concentrations to produce the final library. This was again quantified via Qubit to determine concentration and adjusted to 10 nM concentration with 0.1 M Tris-HCl/0.01% Tween 20 solution prior to sequencing on an Illumina MiSeq platform. Library denaturation and sample loading steps followed the Illumina protocol: sample was loaded at 3pM concentration with 20% PhiX control spike and paired-end sequences generated in 2 × 300 bp format.

### Data Analysis

We created a data analysis pipeline to process the Illumina sequence reads and to match them to known taxa within a local reference database. Files containing the sequence reads used in this study are available through the NCBI sequence read archive (SRA accession PRJNA437768). The source code and tools used for the pipeline are available on github at https://github.com/colford/nbgw-plant-illumina-pipeline. Sequences were quality trimmed and then merged with only sequences greater than 450 bp used in downstream analysis.

A local BLAST database was created from *rbcL* sequence data. This includes reference data for all UK native species^46^ together with sequences from GenBank for non-native species known to be found in the UK. Using this database allowed for unexpected identifications, particularly of non-native species. Each sequence was compared against this database using MegaBlast, and the top 20 maximum bit scores were returned. If these scores matched to a single species, the sequences were assigned to that species. If 60% or more of the sequences matched to a single genus, the sequences were assigned to that genus. BLAST results that did not fall into these two categories were assigned to the category ‘various’.

All results were then checked and verified using expert knowledge. This integrates knowledge of local habitats, species distribution, and rarity to support the BLAST identifications to species and genus and to identify sequences assigned as ‘various’ to family or tribe level where possible. Any remaining sequences blasting to multiple families were classified as ‘unknown’^31,41^.

### Statistical analysis

We converted the number of DNA sequences for each insect to a percentage, to control for differences in DNA amplification between samples in the initial PCR. This can provide semi-quantitative data on the proportions of each pollen taxon^41^. However, we used qualitative data (presence/ absence) for the network analysis and investigation of differences in pollen loads, to avoid any bias caused by differences in pollen retrieval, DNA extraction, amplification and sequencing.

We investigated the pollen transport networks and interspecific differences in pollen loads using two complementary analyses. Interaction network metrics were analysed using the Bipartite Package (v. 2.05) in R version 3.0.1^87,88^, including specialisation (*H*_2_’), which represents the overall level of specialisation of all species in a network, and varies from 0 (complete generalisation) to 1 (complete specialisation); and *d’*, which measures how exclusive a given species’ interactions are compared to the other species in a network, and varies from 0 (no exclusivity) to 1 (completely exclusive).

To test for differences in pollen load composition, we created *a priori* dummy variables representing each comparison arising from our hypothesis predictions (*Cheilosia*/all other genera; *Eristalis*/all other genera; *Rhingia*/all other genera; *Sericomyia*/all other genera; *Volucella*/all other genera). For the species-level analysis of six *Eristalis* species and two *Sericomyia* species (the two genera which had more than one species represented in our samples), comparisons were made within the two genera, and not with other species in this study. We then investigated the similarity between pollen loads between genera and species using the Jaccard similarity index^89^, with statistical differences in pollen loads assessed using *adonis*, a permutational MANOVA procedure in the R package ‘vegan’ version 2.4–3^90^, using 9999 permutations. Since this index is based on species presence - absence, it can overemphasise the significance of rare taxa. To avoid this, we calculated the index based on the data excluding sequences identified to taxa above genus which contributed less than 1% of all sequences. Unidentified sequences were also excluded from the analysis. To correct for multiple comparisons in comparing each genus against all of the other hoverflies, we used the Dunn–Šidák correction. To account for the lack of independence of insects collected within the same site, we used the *strata* argument in *adonis*, which is similar to a random effect in a mixed-effects model.

### Data Availability

The datasets generated and analysed during this study are available from the Dryad repository, 10.5061/dryad.mv0q8v1.

## Electronic supplementary material

Supplementary Information ST1

Supplementary Information ST2

Supplementary Information ST3
